# α-Klotho Expression in Mouse Tissues Following Acute Exhaustive Exercise

**DOI:** 10.3389/fphys.2019.01498

**Published:** 2019-12-10

**Authors:** Zhijian Rao, Lifang Zheng, Hu Huang, Yu Feng, Rengfei Shi

**Affiliations:** ^1^College of Physical Education, Shanghai Normal University, Shanghai, China; ^2^School of Kinesiology, Shanghai University of Sport, Shanghai, China; ^3^Department of Kinesiology and Physiology, East Carolina University, Greenville, NC, United States

**Keywords:** exhaustive exercise, α-klotho, skeletal muscle, liver, adipose tissue

## Abstract

α-Klotho, a multifunctional protein, has been demonstrated to protect tissues from injury via anti-oxidation and anti-inflammatory effects. The expression of α-klotho is regulated by several physiological and pathological factors, including acute inflammatory stress, oxidative stress, hypertension, and chronic renal failure. Exhaustive exercise has been reported to result in tissue damage, which is induced by inflammation, oxidative stress, and energy metabolism disturbance. However, little is known about the effects of exhaustive exercise on the expression of α-klotho in various tissues. To determine the effects, the treadmill exhaustion test in mice was performed and the mice were sacrificed at different time points following exhaustive exercise. Our results confirmed that the full-length (130 kDa) and shorter-form (65 kDa) α-klotho were primarily expressed in the kidneys. Moreover, we found that, except for the kidneys and brain, other tissues primarily expressed the shorter-form α-klotho, including liver, which was in contrast to previous reports. Furthermore, the shorter-form α-klotho was decreased immediately following the acute exhaustive exercise and was then restored to the pre-exercise level or even higher levels in the next few days. Our results indicate that α-klotho may play a key role in the body exhaustion and recovery following exhaustive exercise.

## Introduction

Physical exercise is beneficial for human’s health, including protection from obesity, diabetes, cardiovascular disease, and the delay of aging (Rowe et al., 2014). However, not all types of exercises are good for health. For instance, an exhaustive exercise may damage multiple organs, which is induced by oxidative stress, inflammation, mitochondrial dysfunction, and hormonal changes (Olaìh et al., 2015; Kawanishi et al., 2016; Ke et al., 2016; Liu et al., 2017; Furtado et al., 2018; Yada et al., 2018; Zhang et al., 2019). Certainly, if it is one bout of acute exhaustive exercise, the body can adapt to the imbalances and then recover to the normal state in a few days. However, the exact mechanisms of how exhaustive exercise induces those imbalances and how the body recovers to the normal state are largely unknown.

α-Klotho, an anti-aging protein, was found to be expressed in various tissues in humans and other mammals (Kuro-o et al., 1997) and was reported to play an important role in various organs (Dalton et al., 2017). In addition to its anti-aging effect, α-klotho was demonstrated to be a multifunctional protein that plays roles in energy metabolism, oxidative stress resistance, and anti-inflammation (Xu and Sun, 2015; Dalton et al., 2017; Kuro-o, 2019). Although the expression of α-klotho in some organs is low or absent, α-klotho is still important to maintain the normal function of these organs. For example, a study (Guo et al., 2018) demonstrated that exogenous α-klotho protected the heart form hyperglycemia-induced injury, even though it is well recognized that α-klotho is not expressed in the heart. Sahu et al. (2018) found that α-klotho was upregulated in the young injured muscle, which was important for muscle regeneration and that muscle regeneration could be promoted by the supplement of α-klotho (aa34–981, R&D). Additionally, our previous study found that systemic administration of α-klotho had a pro-lipolytic effect on liver and the white adipose tissue (Rao et al., 2019). Furthermore, α-klotho has been demonstrated to have a protective effect on the heart and kidneys (Guo et al., 2018; Lim et al., 2018). Generally, there are two forms of α-klotho, a full-length (130 kDa) and a shorter form (65 kDa) (Xu and Sun, 2015). The shorter-form α-klotho, also known as soluble α-klotho, is produced when the full-length α-klotho is shed from the cell surface into blood, urine, and cerebrospinal fluid following the proteolytic cleavage of the full-length α-klotho by ADAM10 and ADAM17 (Chen et al., 2007). Following its release from the cell membrane, the circulating shorter-form α-klotho exerts its effects on distant tissues.

The expression of α-klotho was modulated by several physiological and pathological factors, including acute inflammatory stress, oxidative stress, diabetes mellitus, hypertension, and chronic renal failure (Ohyama et al., 1998; Utsugi et al., 2000; Moreno et al., 2011; Asai et al., 2012; Qiu et al., 2016; Poelzl et al., 2018). Also, it has been reported that the serum level of α-klotho was increased following the acute exhaustive exercise training (Sven-Jean et al., 2018), but the source of serum α-klotho was unclear. As we mentioned above, exhaustive exercise is associated with oxidative stress and inflammatory stress in multiple organs. Therefore, it is intriguing to know whether the exhaustive exercise affects the expression of α-klotho in these tissues.

The current study aims to evaluate the expression of α-klotho, as well as to understand the potential function of α-klotho, in various tissues after acute exhaustive exercise.

## Materials and Methods

### Experimental Animals

Eight-week-old C57BL6 male mice were purchased from the Model Animal Research Center (Nanjing, China) and were housed under the controlled temperature and lighting conditions of 20–22°C and 12-h light–darkness cycle. Once the experimental protocol was initiated, all mice were divided into six groups: sedentary (Sed, *n* = 6), immediately after acute exhaustive exercise (E0h, *n* = 7), 12 h after acute exhaustive exercise (E12h, *n* = 7), 24 h after acute exhaustive exercise (E24h, *n* = 7), 3 days after acute exhaustive exercise (E3d, *n* = 7), and 5 days after acute exhaustive exercise (E5d, *n* = 6). Each cage accommodated five mice. All aspects of animal care and experimentation were approved by the Institutional Animal Care and Use Committees of Shanghai University of Sport.

### Exercise Protocol

Before the exercise training, all mice were submitted to the acclimation phase consisting of running on a motorized treadmill at 10 m/min for 5 min, followed by 15 m/min for 5 min and 20 m/min for 10 min at 0% grade every day, consecutively for 3 days. On the day of the experiment, mice were trained with a progressive running paradigm as described by Ma et al. (2018). Briefly, mice were placed on a treadmill, which was followed by their running at a starting speed of 10 m/min for 15 min, at a speed of 15 m/min for 15 min, at a speed of 20 m/min for 15 min, and finally at a speed of 24 m/min until exhaustion. Exhaustion was defined as that the animal’s hindlimbs remained on the electric grid for more than 10 s. No significant difference in the running time to exhaustion was observed among groups.

### Tissue Collection

Mice from each group were euthanized by isoflurane, and the skeletal muscle, including gastrocnemius (Gas), tibialis anterior (TA) and quadriceps (Qua), epididymal white adipose tissue (eWAT) and inguinal white adipose tissue (iWAT), kidneys, brain, lungs, and liver were removed. Samples were immediately flash-frozen in liquid nitrogen until further analysis.

### Quantitative Real-Time PCR

Total RNA from samples was extracted using TRIzol reagent (Invitrogen, Carlsbad, CA, United States) and subjected to quantitative real-time PCR as described (Rao et al., 2019). Gene-specific primer sequences were as follows. For α-klotho, the forward primer was 5′-CACGCCGAGCAAGACTCACTG-3′, and the reverse primer was 5′-TTGATGTCGTCCAACACGTAGGC-3′. For β-actin, the forward primer was 5′-ATCACTATTGGCAACGAGCGGTT C-3′, and the reverse primer was 5′-CAGCACT GTGTTGGCATAGAGGTC-3′. For GAPDH, the forward primer was 5′-AGGCCGGTGCTGAGTATGTC-3′, and the reverse primer was 5′-TGCCTGCTTCACCACCTTCT-3′. Relative gene expressions were calculated with the delta–delta CT method and were normalized with β-actin or GAPDH, using step one plus system (ABI StepOne Plus, Carlsbad, CA, United States).

### Western Blot Analysis

Western blot analysis was performed as described previously (Li et al., 2019). Briefly, lysed samples (40 μg of protein) were separated by SDS-PAGE and transferred to PVDF membranes (Millipore, MA, United States). The membranes were incubated with the antibodies against α-klotho (AF1819, R&D, MN, United States), β-actin (#4970, CST, Beverly, MA, United States), and GAPDH (ab8245, Abcam, Cambridge, United Kingdom). The bands were visualized with enhanced chemiluminescence and quantified by densitometry. The levels of proteins were normalized according to the level of β-actin or GAPDH.

### Statistical Analysis

Data are presented as the mean ±standard error of the mean (SEM). Comparisons among groups were conducted using one-way ANOVA. Data were analyzed using IBM SPSS Statistics 23 (IBM, New York, NY, United States). *P* < 0.05 was denoted as statistically significant.

## Results

### General Characteristics

**FIGURE 1:**
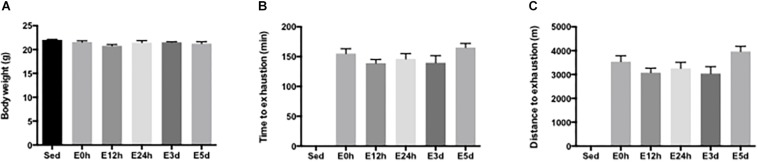
General characteristics of mice. **(A)** Body weight of the mice in each group. **(B)** Running time to exhaustion. **(C)** Running distance to exhaustion. Data are presented as the mean ± SEM (*n* = 6–7 in each group).

The general characteristics of mice, including body weight, time to exhaustion, and distance to exhaustion, were determined. There was no difference in body weight among the groups (Figure 1A). The mean running time to exhaustion for the mice in E0, E12h, E24h, E3d, and E5d groups was 155.0 ± 8.3, 138.9 ± 6.3, 146.0 ± 9.3, 139.8 ± 11.9, and 165.2 ± 7.0 min, respectively (Figure 1B). The mean running distance to exhaustion for the mice in E0, E12h, E24h, E3d, and E5d groups was 3538 ± 253, 3079 ± 188, 3256 ± 258, 3038 ± 293, and 3964 ± 219 m, respectively (Figure 1C). No significant difference in the running time or distance to exhaustion was observed among all groups.

### α-Klotho Expression in Various Tissues

**FIGURE 2:**
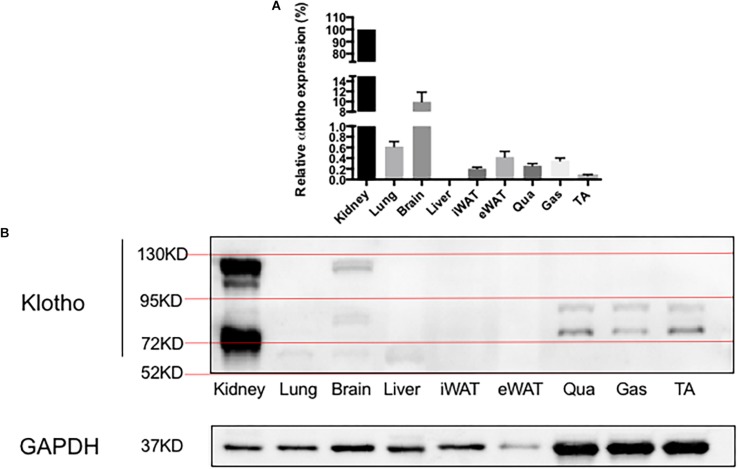
α-Klotho expression in various tissues. **(A)** α-Klotho mRNA expression in various tissues. **(B)** Western blot analysis of GAPDH and α-klotho in the tissues.

α-Klotho mRNA expression levels in various tissues were determined (Figure 2A). The transcripts of α-klotho in the lungs, brain, iWAT, eWAT, Qua, Gas, and TA were 162-, 10-, 491-, 238-, 386-, 286-, and 1372-fold, respectively, and were lower than that in the kidneys. The mRNA expression of α-klotho in the liver was undetectable. α-Klotho protein expression is shown in Figure 2B. We confirmed that α-klotho was primarily expressed in the kidneys. Two forms of α-klotho were found: a 65-kDa protein (actually it was a double band and the molecular weight was about 65–75 kDa) known as the shorter-form α-klotho, and a 130-kDa protein known as the full-length α-klotho. In the brain, besides the shorter-form and full-length α-klotho, a 95-kDa protein was found (actually it was a double band, the molecular weight was about 80 kDa). In the lungs and liver, only the shorter-form α-klotho was observed. However, in the skeletal muscle, two bands, a 75-kDa and a 90-kDa protein, were found. No signal was detected in iWAT and eWAT.

### α-Klotho Expression in the Kidneys After Acute Exhaustive Exercise

**FIGURE 3:**
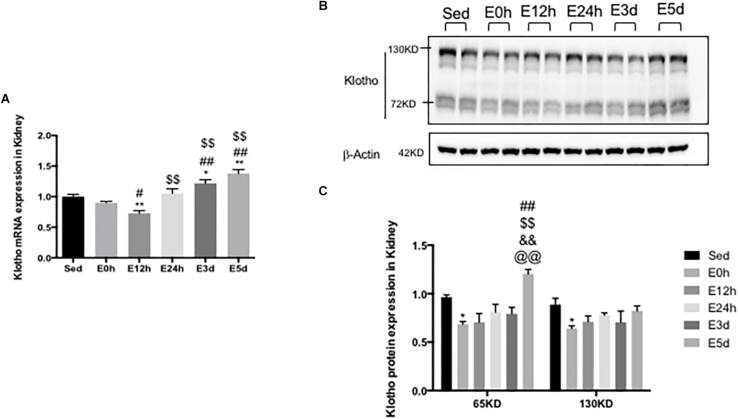
α-Klotho expression in the kidneys following the acute exhaustive exercise. **(A)** mRNA expression of α-klotho (*n* = 6–7 in each group). **(B)** Western blot analysis of actin and α-klotho. **(C)** Quantitative analysis (calculated as ratios of actin densities, *n* = 4 in each group). Results show the relative fold increases over the sedentary group (Sed). Data are presented as the mean ± SEM. ^∗^*P* < 0.05, ^∗∗^*P* < 0.01 vs. Sed group; ^#^*P* < 0.05, ^##^*P* < 0.01 vs. E0h; ^$$^*P* < 0.01 vs. E12h; ^&&^*P* < 0.01 vs. E24h; ^@@^*P* < 0.01 vs. E3d.

To determine the effect of the acute exhaustive exercise on α-klotho expression in the kidneys, α-klotho mRNA (Figure 3A) was analyzed by Q-PCR. In the kidneys, the α-klotho gene expression was decreased at 12 h after the exhaustive exercise (*P* < 0.05), compared to that in the Sed group, and was then restored to the normal level at 24 h after the exhaustive exercise. Three (*P* < 0.05) and five (*P* < 0.01) days after the exhaustive exercise, the α-klotho gene expression levels were significantly increased compared to those in the Sed group. To determine the effect of the acute exhaustive exercise on α-klotho expression in the kidneys, α-klotho protein (Figures 3B,C) was analyzed by Western blot. As shown in Figure 2B, we detected a double band of α-klotho of approximately 65 kDa and a single band of α-klotho of approximately 130 kDa. Both 65-kDa (*P* < 0.05) and 130-kDa (*P* < 0.05) α-klotho protein expression levels were decreased in the mice in the E0 group compared to those in the Sed group. In the mice in the E12h, E24h, and E3d groups, both 65-kDa and 130-kDa α-klotho protein expression levels were in the decreasing trend, compared to those in the Sed group. Interestingly, 5 days after the exhaustive exercise, the 65-kDa α-klotho protein expression level was significantly higher than that in the other four groups except for the Sed group (Figure 3C).

### α-Klotho Expression in the Lung After Acute Exhaustive Exercise

**FIGURE 4:**
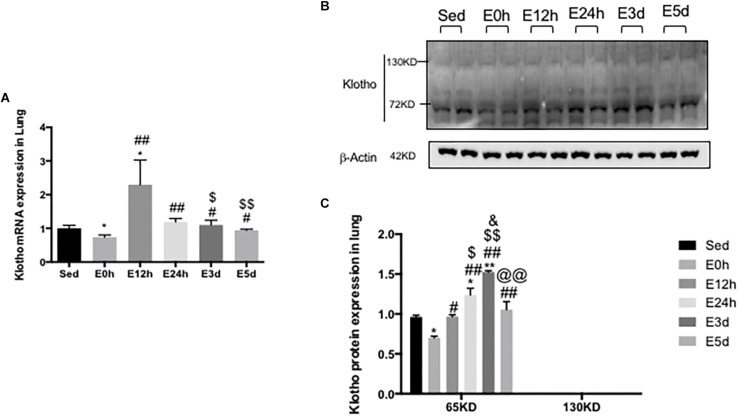
α-Klotho expression in the lungs following the acute exhaustive exercise. **(A)** mRNA expression of α-klotho (*n* = 6–7 in each group). **(B)** Western blot analysis of actin and α-klotho. **(C)** Quantitative analysis (calculated as ratios of actin densities, *n* = 4 in each group). Results show the relative fold increases over the sedentary group (Sed). Data are presented as the mean ± SEM. ^∗^*P* < 0.05 vs. Sed group; ^#^*P* < 0.05, ^##^*P* < 0.01 vs. E0h; ^$^*P* < 0.05, ^$$^*P* < 0.01 vs. E12h; ^&^*P* < 0.05 vs. E24h; ^@@^*P* < 0.01 vs. E3d.

In the lungs, the α-klotho gene expression (Figure 4A) was decreased in the mice in the E0 group, when compared to that in the other five groups (*P* < 0.05 vs. Sed, *P* < 0.01 vs. E12h, *P* < 0.01 vs. E24h, *P* < 0.05 vs. E3d, and *P* < 0.05 vs. E5d), among which the α-klotho gene expression in the mice in the E12h group was significantly increased compared to that in the other four groups (*P* < 0.05 vs. Sed, *P* < 0.01 vs. E24h, *P* < 0.05 vs. E3d, and *P* < 0.01 vs. E5d). As shown in Figure 3B, a single band of α-klotho protein of approximately 65 kDa was observed. The α-klotho protein expression (Figures 4B,C) level was decreased in the mice in the E0 group compared to that in the other five groups (*P* < 0.05 vs. Sed, *P* < 0.05 vs. E12h, *P* < 0.01 vs. E24h, *P* < 0.01 vs. E3d, and *P* < 0.01 vs. E5d). α-Klotho protein expression level was higher in the E24 (*P* < 0.05) and E3d (*P* < 0.01) compared to that in the Sed group (Figure 4C).

### α-Klotho Expression in the Brain After Acute Exhaustive Exercise

**FIGURE 5:**
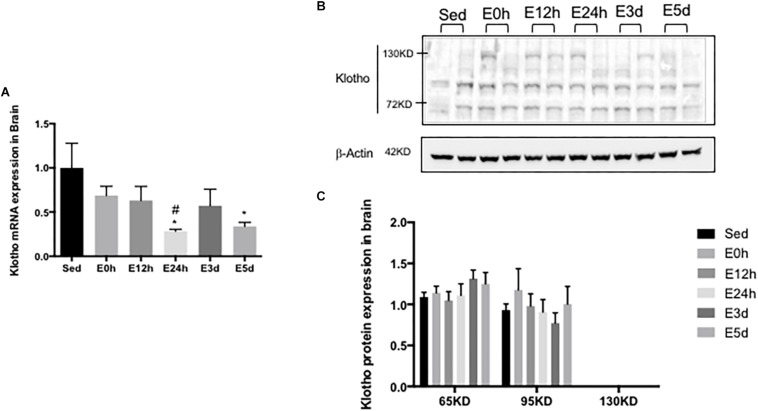
α-Klotho expression in the brain following the acute exhaustive exercise. **(A)** mRNA expression of α-klotho (*n* = 6–7 in each group). **(B)** Western blot analysis of actin and α-klotho. **(C)** Quantitative analysis (calculated as the ratios of actin densities, *n* = 4 in each group). Results show the relative fold increases over the sedentary group (Sed). Data are presented as the mean ± SEM. ^∗^*P* < 0.05 vs. Sed group; ^#^*P* < 0.05 vs. E0h.

In the brain, compared to that in the Sed group, the α-klotho gene expression (Figure 5A) was decreased significantly in the E24h (*P* < 0.05) and E5d (*P* < 0.05) groups. As shown in Figure 5B, our result showed three single bands of α-klotho protein of approximately 65, 95, and 130 kDa, respectively. However, the 130-kDa α-klotho was undetectable in some samples. α-Klotho protein expression level had no changes among the groups (Figure 5C).

### α-Klotho Expression in the Liver After Acute Exhaustive Exercise

**FIGURE 6:**
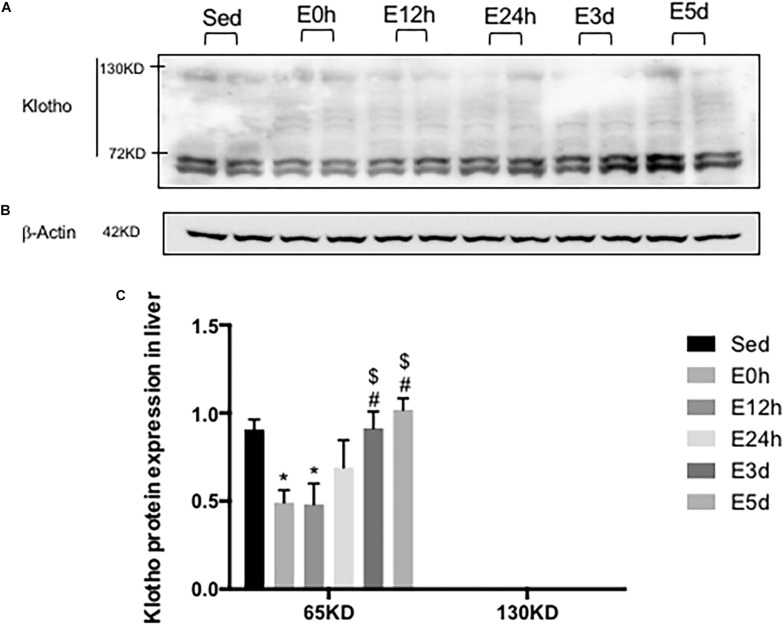
α-Klotho expression in the liver following the acute exhaustive exercise. **(A)** Western blot analysis of α-klotho. **(B)** Western blot analysis of β-actin. **(C)** Quantitative analysis (calculated as the ratios of actin densities, *n* = 4 in each group). Results show the relative fold increases over the sedentary group (Sed). Data are presented as the mean ± SEM. ^∗^*P* < 0.05 vs. Sed group; ^#^*P* < 0.05 vs. E0h; ^$^*P* < 0.05 vs. E12h.

In the liver, the gene expression of α-klotho was undetectable. As shown in Figures 6A,B a double band of α-klotho of approximately 65 kDa was observed. The α-klotho protein expression level was decreased in the E0 (*P* < 0.05) and E12h (*P* < 0.05) groups compared to that in the Sed group. Twenty-four hours after the exercise, the α-klotho protein expression levels were gradually increased, and it was significantly higher in the E3d (*P* < 0.05, *P* < 0.05) and E5d (*P* < 0.05, *P* < 0.05) groups compared to that in the E0h and E12h groups (Figure 6C).

### α-Klotho Expression in the WATs After Acute Exhaustive Exercise

**FIGURE 7:**
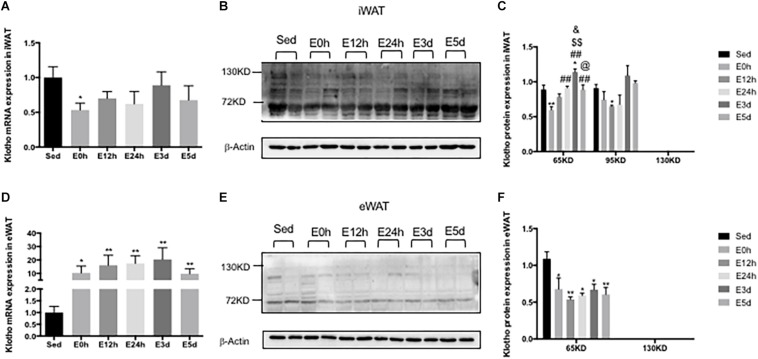
α-Klotho expression in the WATs following the acute exhaustive exercise. **(A,D)** mRNA expression of α-klotho in the iWAT and eWAT (*n* = 6–7 in each group). **(B,E)** Western blot analysis of actin and α-klotho in the iWAT and eWAT. **(C,F)** Quantitative analysis in the iWAT and eWAT (calculated as the ratios of actin densities, *n* = 4 in each group). Results show the relative fold increases over the sedentary group (Sed). Data are presented as the mean ± SEM. ^∗^*P* < 0.05, ^∗∗^*P* < 0.01 vs. Sed group; ^##^*P* < 0.01 vs. E0h; ^$$^*P* < 0.01 vs. E12h; &*P* < 0.05 vs. E24h; @*P* < 0.05 vs. E3d.

In the iWAT, the α-klotho gene expression was decreased immediately after the exhaustive exercise when compared to that in the Sed group (*P* < 0.05), but no changes were found in the other groups (Figure 7A). As shown in Figure 7B, we found two single bands of α-klotho of approximately 65 kDa and 95 kDa in the iWAT. Compared to that in the Sed group, the expression levels of both α-klotho proteins were decreased immediately after the exhaustive exercise (Figure 7C) and were then gradually increased significantly in the E3d group (for 65 kDa α-klotho, *P* < 0.05). In the eWAT, the α-klotho gene expression (Figure 7D) was increased (about 10-fold) after the exhaustive exercise when compared to that in the Sed group (*P* < 0.05 vs. E0h, *P* < 0.01 vs. E12h, *P* < 0.01 vs. E24h, *P* < 0.01 vs. E3d, and *P* < 0.01 vs. E5d). As shown in Figure 7E, in the eWAT, we found one single band of α-klotho of approximately 65 kDa. Compared to that in the Sed group, the α-klotho protein expression levels (Figure 7F) were decreased after the exhaustive exercise (*P* < 0.05 vs. E0h, *P* < 0.01 vs. E12h, *P* < 0.05 vs. E24h, *P* < 0.05 vs. E3d, and *P* < 0.01 vs. E5d).

### α-Klotho Expression in the Skeletal Muscles After Acute Exhaustive Exercise

**FIGURE 8:**
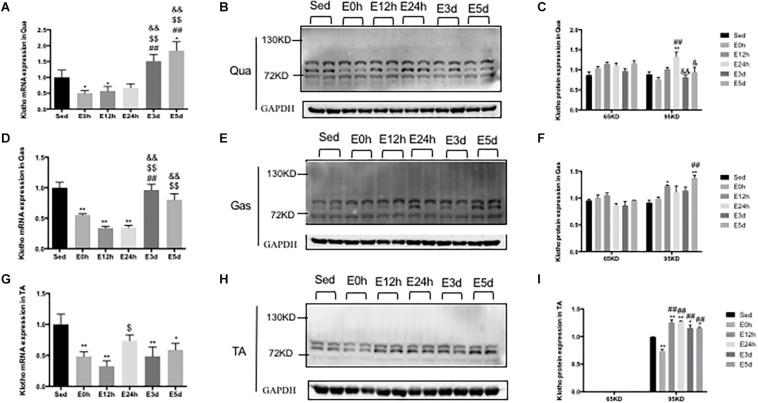
α-Klotho expression in the skeletal muscles following the acute exhaustive exercise. **(A,D,G)** mRNA expression of α-klotho in the Qua, Gas, and TA (*n* = 6–7 in each group). **(B,E,H)** Western blot analysis of GAPDH and α-klotho in the Qua, Gas, and TA. **(C,F,I)** Quantitative analysis in the Qua, Gas, and TA (calculated as the ratios of GAPDH densities, *n* = 4 in each group). Results show the relative fold increases over the sedentary group (Sed). Data are presented as the mean ± SEM. ^∗^*P* < 0.05, ^∗∗^*P* < 0.01 vs. Sed group; ^##^*P* < 0.01 vs. E0h; ^$^*P* < 0.05, ^$$^*P* < 0.01 vs. E12h; ^&^*P* < 0.05, ^&&^*P* < 0.01 vs. E24h.

In the Qua (Figure 8A) and Gas (Figure 8D), compared to that in the Sed group, the α-klotho gene expression was decreased immediately and 12 h after the exhaustive exercise, and was then increased close to or even higher than that in the Sed group. However, in the TA (Figure 8G), the α-klotho gene expression was decreased after the exhaustive exercise, compared to that in the Sed group (*P* < 0.01 vs. E0h, *P* < 0.01 vs. E12h, *P* < 0.01 vs. E3d, and *P* < 0.05 vs. E5d). As shown in Figures 8B,E in the Qua and Gas, we found a single band of α-klotho protein of approximately 65 kDa and a double band of α-klotho protein of approximately 95 kDa. However, as shown in Figure 8H, in the TA, our result showed a double band of α-klotho of approximately 95 kDa. For the 65-kDa α-klotho protein expression, no significant change was found in the skeletal muscle among the groups (Figures 8C,F,I). For the 95-kDa α-klotho protein, the α-klotho protein expression level tended to increase following the acute exhaustive exercise in the Qua (Figure 8C) and Gas (Figure 8F), when compared to that in the Sed group. However, in the TA (Figure 8I), the α-klotho protein expression level was decreased immediately after the exhaustive exercise, and was then increased rapidly, to the level that was even significantly higher than that in the Sed group (*P* < 0.01 vs. E12h, *P* < 0.01 vs. E24h, *P* < 0.05 vs. E3d, and *P* < 0.05 vs. E5d).

## Discussion

The anti-aging protein α-klotho, first reported by Kuro-o et al. (1997), is mainly expressed in a number of specific tissues including kidneys, skeletal muscle, urinary bladder, aorta, pancreas, testis, ovary, colon, and thyroid gland, whereas the mRNA expression of the protein was not detected in the stomach, lungs, heart, liver, skin, and bone. Using immunohistochemistry (Ab69208 and Ab181373, Abcam), Li et al. (2019) confirmed that α-klotho was expressed in human’s kidneys, arterial tree, epithelia, neural tissues, thyroid, pancreas, testis, and prostate, but not in the liver. Consistently, we detected the full-length α-klotho (130 kDa) and shorter-form α-klotho (65 kDa) in the kidneys.

It has been reported that neither α-klotho mRNA nor protein is expressed in murine, rat, and human liver (Kuro-o et al., 1997; Sato et al., 2015; Yamauchi et al., 2016). However, when more sensitive methods were used, the transcript of α-klotho in the liver was found to be about 10,000-fold lower than that in the kidneys (Madathil et al., 2014). Another study also reported that the α-klotho protein was expressed in liver tissues and HCC cells (Shu et al., 2013). In the current study, while α-klotho mRNA was undetectable by RT-PCR, a low level of full-length α-klotho protein was expressed in the liver, as illustrated by the faint band on the Western blot. We also detected the shorter form of α-klotho protein in the liver (Figure 3B). However, it is not clear whether the shorter form of α-klotho was expressed in the liver locally or whether it was the shed form of the protein that came from other tissues such as kidneys. Furthermore, no study has reported that α-klotho plays any roles in the liver so far, except our previous study that found that α-klotho might have a pro-lipolysis effect in the liver in obese mice (Rao et al., 2019). In contrast, the α-klotho homolog, β-klotho, was reported to be expressed in the liver, where it appeared to have a role in glucose metabolism (Kurosu et al., 2007).

It was originally reported that α-klotho mRNA was not detected in murine, rat, and human lungs (Kuro-o et al., 1997; Ohyama et al., 1998). But later studies indicated that α-klotho was expressed in the lungs at a very low level (Sato et al., 2015; Yamauchi et al., 2016). Indeed, we detected both α-klotho mRNA and protein in the lungs. Notably, the mRNA expression level of α-klotho in the lungs was 162-fold lower than that in the kidneys. We also found a faint band of full-length α-klotho and a clear band of the shorter-form α-klotho in the lungs using Western blot, demonstrating that the α-klotho protein did express in the lungs. In the lungs, α-klotho may indeed be expressed at a very low level, but studies of lung tumor samples have demonstrated that loss of α-klotho expression is associated with lower survival rates (Usuda et al., 2011a, b). As in the case of liver, the source of shorter-form α-klotho protein in the lungs is unclear and further study is needed.

The brain was initially demonstrated to express a high level of α-klotho mRNA in both mouse and human (Kuro-o et al., 1997). Later studies found that several different areas in the brain expressed α-klotho, including cerebellar Purkinje cells, hypothalamus, thalamus, striatum, and cerebellum (Degaspari et al., 2015; Dubal et al., 2015; Lim et al., 2015). In the current study, however, the mRNA expression level of α-klotho in the brain was approximately 10-fold lower than that in the kidneys. The protein expression of α-klotho in the brain was detected mainly by IHC (German et al., 2012; Clintona et al., 2013; Brobey et al., 2015; Lim et al., 2015). Xin et al. (2015) determined that α-klotho was expressed in the brain using Western blot (MAB1819, R&D), but they did not report the size of the molecule. Our data showed that there are three kinds of α-klotho protein whose molecular sizes are 65, 95, and 130 kDa. It is well known that α-klotho has a membrane form (the full-length) and a shed form (the shorter form) (Matsumura et al., 1998). However, our Western blots detected three bands with corresponding molecular sizes of 65, 95, and 130 kDa, respectively. We are the first to report the 95-kDa band detected by anti-α-klotho antibodies. Future study is needed to determine whether it is non-specific or represents a new form of α-klotho. Additionally, the 130-kDa α-klotho protein is undetectable in some brain samples; it might result from the fact that we did not select any particular areas of the brain when we prepared samples for Western blot. Together, the results indicate that not all neuron cells express the full-length α-klotho protein.

Interestingly, we also found three forms of α-klotho protein in the iWAT (Figure 6B), but only two forms of α-klotho protein in the eWAT (Figure 7E). Notably, the expression level of the full-length of α-klotho protein in the WATs (both iWAT and eWAT) was very low. Chihara et al. (2006) also detected the full-length of α-klotho in the adipocyte cell line using Western blot (KM2076, TransGenic). However, the results were controversial (Lorenzi et al., 2010; Stuelsatz et al., 2012). In the current study, we demonstrated that the α-klotho protein was expressed in the WATs, although the expression level was very low. The study of the function of α-klotho in the adipose tissues is limited. Chihara et al. found that α-klotho played an essential role in adipocyte differentiation (Chihara et al., 2006), and our previous study determined that α-klotho had a pro-lipolysis function in the white adipose tissue under the obese condition (Rao et al., 2019). Future study is needed to confirm the lipolysis function of α-klotho, because it is important to know whether it is possible to enhance the endurance performance by supplementing α-klotho.

α-Klotho expression level in the skeletal muscle was thought to be intermediate (Kuro-o et al., 1997). But subsequent studies indicated that α-klotho expressed in murine, rat, and human skeletal muscle was very low or absent (Matsumura et al., 1998; Ohyama et al., 1998; Yamauchi et al., 2016). Our data showed that the mRNA expression level of α-klotho in the skeletal muscles was 286- to 1372-fold lower than that in the kidneys. Of note, the full-length α-klotho protein was not found in any skeletal muscle; 65-kDa and 95-kDa α-klotho proteins were found in the Qua, Gas, and TA, but the expression of the 65-kDa α-klotho protein was low in the TA. In contrast, Wehling-Henricks et al. (2016) showed that the full-length α-klotho protein was expressed in the skeletal muscle using Western blot (ab75023, Abcam). The conflicting results are most likely due to the difference in the antibodies used. Studies also determined that α-klotho protein exists in the skeletal muscle using IHC (Sahu et al., 2018). The current study was the first to provide the evidence that the shorter-form (but not in TA) and 95-kDa α-klotho protein were expressed in the skeletal muscle. The forms of α-klotho protein may be different in different types of skeletal muscle. As shown in our study, the shorter-form α-klotho protein did not exist in the fast muscle (TA), whereas the 95-kDa α-klotho protein existed in all types of skeletal muscle. Further study is needed to determine why the distribution of α-klotho protein isoforms occurs.

The expression of α-klotho is regulated by many factors. Exercise (both acute and chronic) was reported to affect the expression of α-klotho in the brain and kidneys (Ji et al., 2018), as well as the level of soluble α-klotho in the serum (Matsubara et al., 2014; Santos-Dias et al., 2016; Dalise et al., 2017; Amaro-Gahete et al., 2019). But the physiological mechanism is unclear. Furthermore, little is known about the relationship between α-klotho and acute exhaustive exercise. To our knowledge, the current study is the first study to investigate the effects of the acute exhaustive exercise on α-klotho expression in various tissues. Our results showed that α-klotho expression (in most of the organs except the brain) was decreased immediately after the acute exhaustive exercise, and then restored to the pre-exercise or even higher level in next 12–24 h. Oxidative stress injury was induced in various tissues during the exhaustive exercise and was removed during the recovery (Ke et al., 2016; Zhang et al., 2019), and it has also been demonstrated that α-klotho expression would be reduced by oxidative stress (Mitobe et al., 2005; Acikgoz et al., 2006). Therefore, exhaustive exercise-induced oxidant stress injury may result in reduced α-klotho expression in various tissues. Interestingly, acute exhaustive exercise was reported not to cause significant oxidative stress in the brain during the post-exercise period (Acikgoz et al., 2006), which might be the explanation of the unaltered α-klotho expression in the brain after the acute exhaustive exercise in the current study. Of note, the shorter-form α-klotho (65 or 95 kDa) was the main α-klotho protein that existed in most tissues (except the kidneys). These results suggest that exhaustive exercise-induced exhaustion might be associated with the decreased expression level of the shorter-form α-klotho, and the upregulated level of shorter-form α-klotho might contribute to the recovery of the body from exhaustion. Thus, future study is needed to further investigate whether a supplement of α-klotho can alleviate exhaustive exercise-induced injury, promote recovery, and even enhance exercise performance.

Taken together, we demonstrated that most tissues (except the kidneys) mainly expressed shorter-form α-klotho (65 kDa or 95 kDa), which was decreased immediately after acute exhaustive exercise and was then restored to the pre-exercise or even higher levels in the next few days. Our results indicate that α-klotho might play a key role in the body exhaustion and recovery following exhaustive exercise.

## Data Availability Statement

All datasets generated for this study are included in the article/supplementary material.

## Ethics Statement

The animal study was reviewed and approved by the Institutional Animal Care and Use Committees of Shanghai University of Sport.

## Author Contributions

ZR and LZ performed all the experiments and analyzed the data. YF and LZ helped to maintain the experimental mice. ZR, RS, and HH designed the experiments and wrote the manuscript. All authors reviewed and approved the submission of the manuscript.

## Conflict of Interest

The authors declare that the research was conducted in the absence of any commercial or financial relationships that could be construed as a potential conflict of interest.
